# Photothermal-Assisted Solvent-Free Decontamination of a Nerve Agent Simulant Using UiO-66-NH_2_@CNT Hybrids

**DOI:** 10.3390/nano16110690

**Published:** 2026-06-01

**Authors:** Haechan Cho, Jonghyeok Bang, Seungheon Oh, Jinyoung Chung, Ji Won Lim, Heesoo Jung, Youngho Jin

**Affiliations:** 1 Department of Advanced Materials Engineering, Chung Ang University, 4726, Seodong-daero, Daedeok-myeon, Anseong-si 17546, Republic of Korea; gocks9570@cau.ac.kr (H.C.); hyki119@cau.ac.kr (J.B.); tmdgus0150@cau.ac.kr (S.O.); 2 Pharmcle Co., Ltd., 10-gil, Seolleung-ro, Gangnam-gu, Seoul 06194, Republic of Korea; cjy@pharmcle.com; 3 ADD Agency for Defense Development, Daejeon 34060, Republic of Korea; ljw@add.re.kr

**Keywords:** UiO-66-NH_2_, photothermal hybrids, solvent-free decontamination, chemical warfare agents, reactive adsorption

## Abstract

Chemical warfare agents (CWAs) pose severe threats to human health and the environment because of their extreme toxicity. Conventional liquid-phase decontamination processes can present limitations, including potential equipment corrosion, generation of secondary liquid waste, and increased operational complexity. To overcome these challenges, we report a solar-assisted process intensification strategy for solvent-free decontamination of toxic organophosphorus compounds using UiO-66-NH_2_@carbon nanotube (CNT) hybrid platforms. Incorporation of CNTs (optimized at 5 wt%) enables efficient solar-to-thermal conversion, resulting in rapid photothermal self-heating to 85 °C under simulated solar irradiation (1000 W m^−2^). This localized thermal effect contributes to accelerated DMMP removal within the MOF-based hybrid structure, thereby partially alleviating the kinetic limitations typically associated with solvent-free reactions. Consequently, the optimized hybrid achieves 94% removal of dimethyl methylphosphonate (DMMP), a representative sarin simulant, within 10 min under humidity-conditioned, solvent-free conditions, representing a 27% improvement compared with pristine UiO-66-NH_2_. This decontamination platform eliminates the need for chemical solvents and external energy input, thereby mitigating secondary contamination and reducing the environmental footprint. By integrating the catalytic framework of Zr-based MOFs with the photothermal capability of CNTs, this study presents a sustainable engineering strategy for advanced defense and environmental protection.

## 1. Introduction

Chemical warfare agents (CWAs) are among the most hazardous toxic chemicals because of their extreme toxicity and rapid dissemination potential, posing severe risks to both military operations and civilian safety [1,2,3]. Effective defense in real-world scenarios, therefore, requires potentially field-deployable and sustainable decontamination platforms capable of rapidly neutralizing these agents without external power input or liquid solvents [4,5,6]. Traditional liquid-phase decontamination methods, although widely used, suffer from significant limitations, including equipment corrosion [7], generation of hazardous secondary waste [8,9] and logistical constraints in solvent-limited military environments [10].

Metal–organic frameworks (MOFs) have emerged as leading candidates for CWA decontamination owing to their exceptionally high surface areas, tunable surface architecture, and versatile surface-active metal sites [11,12,13,14,15,16]. Among them, the UiO-66 series is particularly notable for its outstanding chemical and thermal stability, which originates from its robust Zr_6_O_4_(OH)_4_ nodes [17,18]. Amine-functionalized UiO-66-NH_2_ has attracted considerable attention because its dual functional sites, Lewis-acidic Zr-oxo clusters and nucleophilic amine groups are believed to contribute to adsorption and surface-mediated transformation of organophosphorus compounds [19,20,21,22,23]. The structural tunability and light-responsive properties of these MOF-based platforms further enable integration of solar-driven functionalities [24], thereby extending their applications to environmental remediation [25,26,27], hazardous chemical detection [28,29], and advanced membrane separation technologies [30,31,32].

Despite these advantages, a significant bottleneck remains in translating MOF-based catalysis from laboratory-scale liquid-phase systems to practical solvent-free applications. Under solvent-free conditions, the decontamination efficiency of MOFs is often severely limited by sluggish reaction kinetics and mass-transfer constraints at the solid-phase interface [33,34]. Most reported systems still operate in aqueous environments, where performance is influenced by ambient moisture and where efficient diffusion pathways are lacking [35,36]. Therefore, developing a strategy capable of overcoming these kinetic and diffusion barriers in a solvent-free state is essential for next-generation defense technologies.

Photothermal intensification provides a sustainable and efficient approach by converting abundant solar energy into localized thermal energy, thereby accelerating catalytic reactions at the molecular level [24,37,38]. Carbon nanotubes (CNTs) are particularly suitable photothermal transducers because of their broadband light absorption [39], rapid thermal response, and superior thermal conductivity relative to conventional hybrid components [40,41,42]. By engineering the interface between the photothermal CNT network and the catalytic MOF domains [43], a localized hot-spot effect can be generated to drive rapid chemical transformations even in the absence of solvents.

In this study, we present a powder-form decontamination platform based on UiO-66-NH_2_@CNT hybrids for solvent-free removal of dimethyl methyl phosphonate (DMMP), a sarin simulant. By tuning the CNT loading, a reasonable balance between solar-to-thermal conversion and accessibility of catalytic active sites is achieved [44,45,46,47]. Under simulated solar irradiation (1000 W m^−2^), the hybrid exhibits rapid self-heating to 85 °C, enabling approximately 94% DMMP removal within 10 min under solvent-free conditions [38,48].

Rather than introducing an entirely new photothermal detoxification concept, this work demonstrates how CNT-assisted photothermal heating can accelerate early-stage DMMP removal kinetics in a simple powder-form MOF hybrid system. This solvent-free decontamination strategy may offer potential utility in defense and protective material applications [49,50,51]. The powder-form hybrid system may be adaptable for integration into protective textiles and military infrastructure [40,52,53]. Furthermore, by eliminating the logistical burden associated with liquid-based systems, this approach offers a sustainable alternative that reduces the environmental footprint in resource-limited environments [54,55,56]. Overall, this work establishes a promising material design framework for multifunctional materials for chemical safety [57], providing a technical foundation for next-generation defense technologies [58].

**Figure 1:**
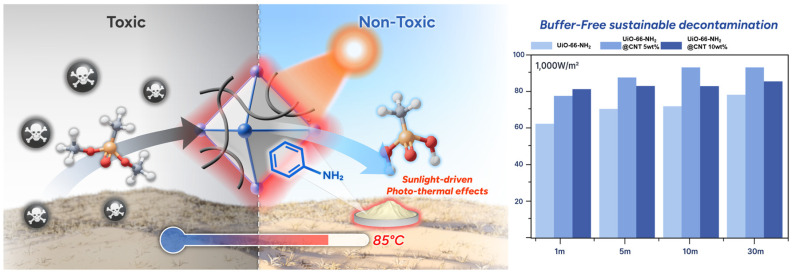
Conceptual illustration of solar-driven surface activation in UiO-66-NH_2_@CNT hybrid powders for sustainable solvent-free removal of organophosphorus simulants. The hybrid integrates the Lewis-acidic surface-active sites of UiO-66-NH_2_ with the photothermal heating capability of CNTs, enabling rapid, solvent-free decontamination under simulated sunlight. The schematic represents the proposed photothermal-assisted reactive adsorption process.

A conceptual illustration of the proposed photothermal-assisted solvent-free decontamination mechanism is presented in Figure 1.

## 2. Materials and Methods

### 2.1. Materials

Zirconium(IV) chloride (ZrCl_4_, 97%, Sigma-Aldrich, St. Louis, MO, USA), 2-aminoterephthalic acid (NH_2_BDC; 99%, Sigma-Aldrich), N,N-dimethylformamide (DMF, 99.0%, Duksan General Science Co., Seoul, Republic of Korea), acetic acid (99.7%, Duksan General Science, Republic of Korea), hexane, tributyl phosphate (TBP), acetone, CNTs (99 wt%, Carbon Nano-material Technology Co., Pohang, Republic of Korea), and DMMP (Sigma-Aldrich; a simulant for GD) were used as received. Decontaminant components were also employed as provided.

### 2.2. Synthesis Procedures

UiO-66-NH_2_: ZrCl_4_ (200 mg, 0.858 mmol) and NH_2_BDC (191 mg, 1.143 mmol) were dispersed in DMF (20 mL) by sonication for 30 min, after which acetic acid (8.88 mL, 9.315 g, 155 mmol) was added. The mixture was subjected to microwave heating at 150 °C for 30 min. The microwave power given for the synthesis experiment is 150 W initially to elevate the temperature environment to 150 °C. After it reaches its desired temperature, the system reduces the power to 30~40 W to maintain the inner temperature environment till the controlled time. The resulting solid was collected and washed five times with DMF (10 mL per wash), followed by five washes with acetone (10 mL per wash). The product was then dried at 120 °C overnight and subsequently ground using a ball mill to obtain fine UiO-66-NH_2_ powder.

UiO-66-NH_2_@CNT: CNTs (5 or 10 wt%) were first dispersed in DMF (20 mL) by ultrasonication for 1 h. In parallel, ZrCl_4_ (200 mg, 0.858 mmol) and NH_2_BDC (191 mg, 1.143 mmol) were dispersed in DMF (20 mL) by sonication for 30 min. The CNT dispersion was combined with the precursor solution, followed by the addition of acetic acid (8.88 mL, 9.315 g, 155 mmol). The resulting mixture was heated under microwave irradiation at 150 °C for 30 min. The solid product was collected and washed five times with DMF (10 mL per wash) and five times with acetone (10 mL per wash), then dried at 120 °C overnight. Finally, the dried composite was ball-milled to obtain fine UiO-66-NH_2_@CNT (5 wt% or 10 wt%) powders. For simplicity, UiO-66-NH_2_@CNT composites are hereafter abbreviated as UiO@CNT-x wt%, where x represents the CNT loading.

### 2.3. Decontamination Experiments

In the decontamination experiments, DMMP was selected as a representative G-series nerve agent simulant because of its structural similarity to organophosphorus nerve agents [12]. Prior to testing, the synthesized samples were dried under vacuum for 1 h and subsequently equilibrated at 70% relative humidity for 24 h to simulate realistic atmospheric exposure conditions. Additional thermogravimetric analysis (TGA) was performed to verify the presence of physically adsorbed water introduced during the humidity-conditioning process (Figure S1). For each experiment, 5 mg of the sample was placed in a 2 mL vial. All decontamination experiments were performed using the same total mass of composite material to evaluate the influence of CNT incorporation on the overall photothermal-assisted decontamination behavior. DMMP was diluted in hexane (5 mg mL^−1^), and 20 μL of the simulant solution was introduced into each vial to ensure uniform delivery of the simulant onto the solid surface. Hexane was used only as a volatile carrier solvent and was expected to rapidly evaporate after application, allowing the reaction to proceed predominantly under humidity-conditioned, solvent-free conditions without the addition of bulk liquid water. The mixture was vortex-mixed for 30 s to ensure uniform contact between the simulant and the solid material. After the desired reaction time, ethyl acetate (EA) was added to quench the reaction, and tri-n-butyl phosphate (TBP) was used as an internal standard for GC analysis. The vial was tightly sealed and kept stationary for 1 h to extract residual DMMP species adsorbed within the porous structure of UiO-66-NH_2_. The extracted solution was filtered using a syringe and transferred to a clean vial for GC–FID analysis. The decontamination efficiency was quantitatively determined from the decrease in the DMMP peak area based on a pre-established calibration curve. After the initial decontamination cycle, the used samples were recovered and dried at 120 °C for 2 h to remove residual species, then reused under identical conditions to evaluate their regeneration capability.

### 2.4. Photothermal Effect Testing

Photothermal experiments were conducted using a lamp power system (DY-Tech Co., Seoul, Republic of Korea) providing a broad emission spectrum from 350 to 1100 nm. The lamp output power was adjustable from 0 to 500 W, and the irradiation distance was controlled using a height-adjustable rack. The light intensity at the sample position was measured with a solar power meter (TES-132, 0–200 W m^−2^) before testing, and irradiation intensities of 500 and 1000 W m^−2^ were selected for subsequent decontamination experiments.

Ultraviolet–visible (UV–Vis) spectroscopy and photothermal performance measurements were performed using composite films prepared with polydimethylsiloxane (PDMS) as the matrix. Each sample was incorporated into PDMS at a designated loading ratio (x) and homogenized using a Thinky ARE-310 planetary mixer (Thinky Co., Tokyo, Japan) at 1000 rpm for 5 min. The mixture was then poured into a 35 mm Petri dish and cured to obtain a uniform film for optical characterization. Photothermal heating measurements related to the decontamination experiments were conducted separately using powder-form samples under irradiation conditions.

### 2.5. Physical Measurements

The crystalline structures of UiO-66-NH_2_ and its CNT composites were characterized by X-ray diffraction (XRD) using a Bruker D8 DISCOVER diffractometer (Bruker Co., Billerica, MA, USA). Transmission electron microscopy (TEM) was performed to examine the morphology and interfacial structure of UiO-66-NH_2_ and UiO-66-NH_2_@CNT composites using a JEOL JEM-2100 (JEOL Ltd., Tokyo, Japan) operated at an accelerating voltage of 200 kV at the Central Research Facility, Chung-Ang University. X-ray photoelectron spectroscopy (XPS; Thermo Scientific K-Alpha Plus, Thermo Fisher Scientific, Waltham, MA, USA) was conducted to characterize the surface electronic states of UiO-66-NH_2_ and its CNT composites. The spectra indicated preservation of the UiO-66-NH_2_ coordination environment and the formation of interfacial UiO–CNT interactions. CNT incorporation introduced an amide-related O 1s component and increased C–N contributions, while the framework structure remained stable up to 10 wt% CNT loading. Attenuated total reflectance Fourier transform infrared (ATR-FTIR) spectra were collected using a Nicolet i50 spectrometer (Thermo Fisher Scientific, Waltham, MA, USA) equipped with a DiffusIR™ accessory (PIKE Technologies, Madison, WI, USA) over the range of 400–4000 cm^−1^. UV–Vis diffuse reflectance spectra were recorded with a Shimadzu UV-2600i spectrophotometer (Shimadzu Co., Kyoto, Japan) to evaluate the light-absorption properties of the materials. Scanning electron microscopy (SEM) was performed using a SIGMA 360 (FE) (Carl Zeiss AG, Oberkochen, Baden-Württemberg, Germany) instrument operated at accelerating voltages ranging from 1 to 50 kV.

Nitrogen adsorption–desorption isotherms were measured at 77 K using an automated volumetric adsorption analyzer (Micromeritics TriStar 3020, Micromeritics Instrument Co., Norcross, GA, USA) to determine the pore structure and Brunauer–Emmett–Teller (BET) surface area. Before analysis, the samples were degassed under vacuum at 80 °C for 1 h, followed by heating to 150 °C for 2 h. The photothermal responses of UiO-66-NH_2_, UiO-66-NH_2_@CNT 5 wt%, and UiO-66-NH_2_@CNT 10 wt% were evaluated using a lamp irradiation system (DY-Tech, Republic of Korea) under simulated light intensities of 500 and 1000 W m^−2^. Surface temperature variations were monitored in real time using a forward-looking infrared (FLIR) thermal camera, and representative thermal images were recorded at selected time intervals. Pyridine-adsorbed Fourier transform infrared (Py-IR) spectra were recorded using a Nicolet i50 spectrometer (Thermo Scientific). Before measurement, the samples were preheated to remove physisorbed moisture, then exposed to pyridine vapor at 100 °C for 1 h to ensure adsorption. Subsequently, the samples were evacuated to remove weakly adsorbed pyridine before spectral acquisition. Spectra were collected over the range of 400–4000 cm^−1^ to probe the accessibility of Lewis acidic sites via pyridine adsorption.

## 3. Results

The photothermal-assisted decontamination performance of the UiO-66-NH_2_@CNT hybrid was evaluated under solvent-free conditions. To contextualize the advantages of this solvent-free powder system, its performance was compared with representative MOF-based decontamination systems reported in the literature.

As summarized in Table 1, previous MOF-based systems have demonstrated efficient decontamination of CWA simulants under various conditions, including fabric-supported platforms and moisture-assisted environments. Rather than directly competing with these approaches, the present work introduces a distinct operational concept based on a powder-form and solvent-free reactive adsorption system for practical decontamination.

In contrast to conventional configurations that rely on external media or substrate supports, the UiO-66-NH_2_@CNT (5 wt%) hybrid developed here achieves ≈ 94% DMMP decontamination within 10 min under buffer-free conditions. This strategy integrates adsorption, catalytic transformation, and photothermal self-heating within a single material platform, enabling efficient decontamination without additional solvents or external heating sources.

These features demonstrate the practical and environmentally sustainable advantages of this powder-form system and underscore its potential for field-relevant decontamination of toxic agents.

**Figure 2:**
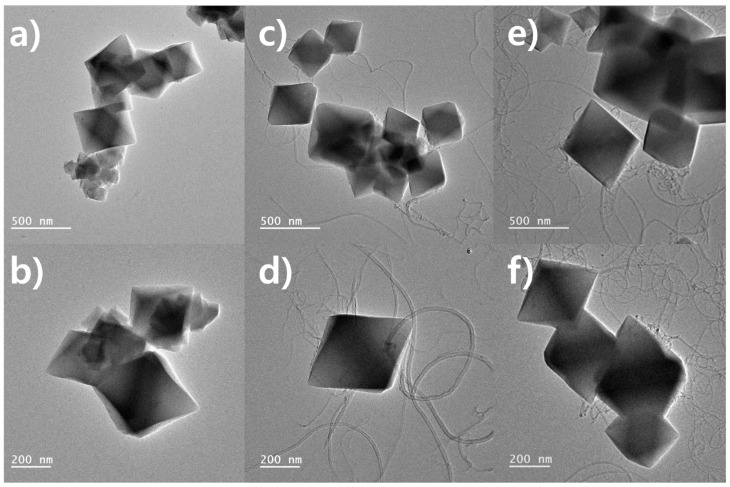
TEM images of (**a**,**b**) UiO-66-NH_2_, (**c**,**d**) UiO-66-NH_2_@CNT 5 wt%, and (**e**,**f**) UiO-66-NH_2_@CNT 10 wt%. Pristine UiO-66-NH_2_ exhibits uniform octahedral crystals, whereas CNT incorporation results in intimate MOF–CNT hybrids without structural collapse.

The TEM images of UiO-66-NH_2_ particles in Figure 2a,b display well-defined octahedral crystals with smooth facets and a relatively uniform size of approximately 200–400 nm, consistent with the formation of highly crystalline UiO-66-NH_2_. In the TEM images of the UiO-66-NH_2_@CNT (5 wt%) composite shown in Figure 2c,d, CNT strands are observed in contact with the MOF crystals, suggesting partial interfacial association while preserving the octahedral morphology. Such intimate interfacial contact is expected to facilitate localized heat transfer across the MOF–CNT interface, which is beneficial for photothermal processes [40,41]. Similarly, the TEM images of the UiO-66-NH_2_@CNT (10 wt%) composite in Figure 2e,f show that, although a denser CNT network partially covers the MOF surface, the overall octahedral morphology of the UiO-66-NH_2_ particles is retained without obvious structural collapse, indicating successful incorporation of CNTs.

**Figure 3:**
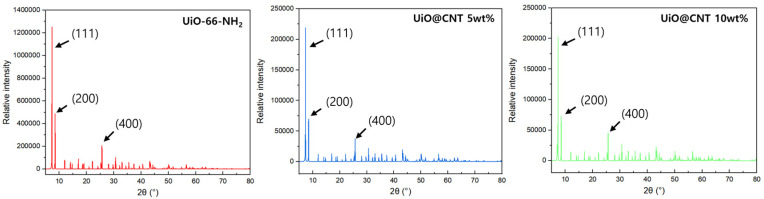
XRD patterns of pristine UiO-66-NH_2_, UiO-66-NH_2_@CNT 5 wt%, and UiO-66-NH_2_@CNT 10 wt%. All samples exhibit characteristic reflections corresponding to the (111), (200), and (400) planes, indicating preservation of the crystalline MOF after CNT incorporation.

The XRD patterns are presented in Figure 3. All samples exhibit characteristic diffraction peaks at 2θ = 7.4° and 8.6°, corresponding to the (111) and (200) planes of the UiO-66-type framework [17], confirming the formation of crystalline UiO-66-NH_2_ [36]. In addition, a diffraction feature at approximately 26° is observed, which may arise from overlapping contributions of the (400) plane of the UiO-66 framework and the characteristic (002) reflection of CNTs. After incorporation of CNTs, the positions of the main diffraction peaks remain unchanged, with slightly reduced intensities, indicating that the intrinsic MOF is preserved during composite formation.

No additional diffraction peaks indicative of impurity phases are detected, confirming that the CNT incorporation process preserves the phase purity and crystalline integrity of UiO-66-NH_2_. The elemental mapping results in Figure S2 further support this conclusion, showing uniform distributions of Zr and O along with well-dispersed CNTs, indicating preservation of the framework structure and homogeneous spatial coexistence of both components.

**Figure 4:**
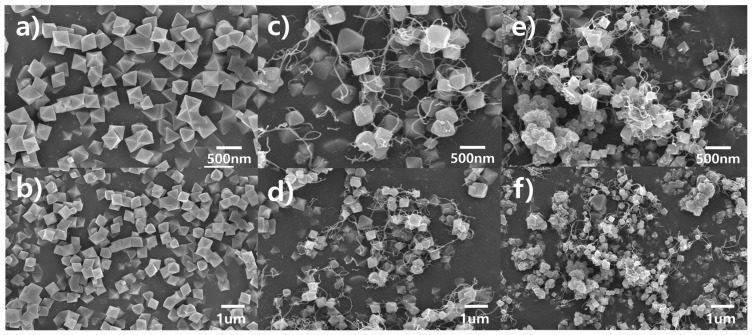
SEM images of (**a**,**b**) UiO-66-NH_2_, (**c**,**d**) UiO-66-NH_2_@CNT 5 wt%, and (**e**,**f**) UiO-66-NH_2_@CNT 10 wt%.

The morphologies of UiO-66-NH_2_ and the UiO-66-NH_2_@CNT composites, as revealed by SEM, indicate that UiO-66-NH_2_ exhibits uniform octahedral crystals with edge lengths of 200–400 nm (Figure 4a,b). The UiO-66-NH_2_@CNT (5 wt%) composite shows a similar crystal size (200–400 nm), accompanied by a more open particle arrangement and well-dispersed CNT networks bridging adjacent MOF particles (Figure 4c,d). In contrast, UiO-66-NH_2_@CNT (10 wt%) displays partial aggregation, forming clustered structures with sizes of approximately 300–800 nm (Figure 4e,f), which may limit the accessibility of active surface sites. These observations indicate that moderate CNT loading preserves the overall MOF morphology while introducing interparticle CNT networks, whereas higher CNT content promotes aggregation, consistent with previous reports [47,60]. In addition, thermogravimetric analysis (TGA) confirms that CNT incorporation increases the residual mass and apparent thermal robustness of the composites (Figure S3).

**Figure 5:**
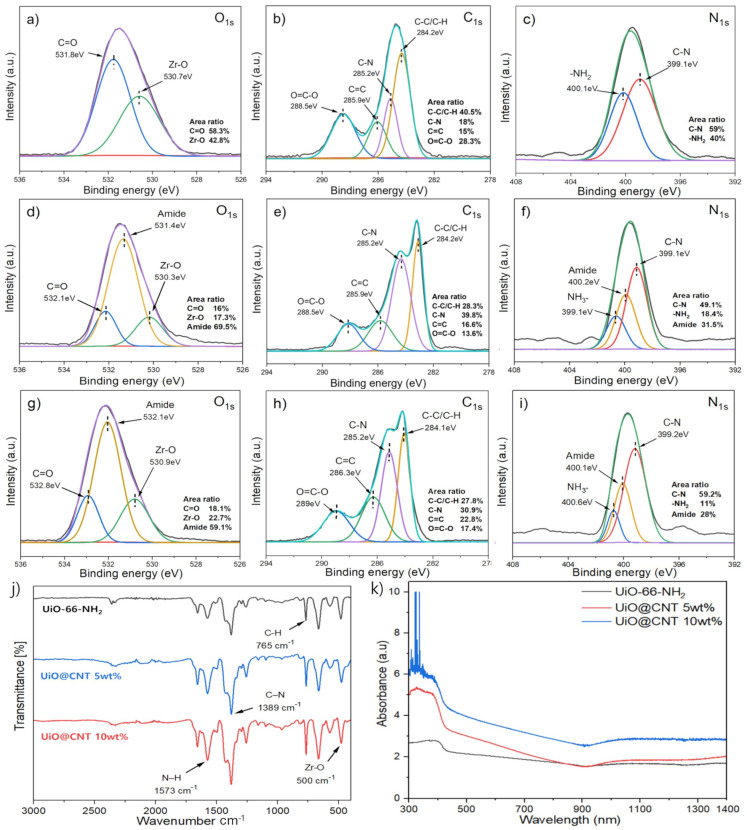
(**a**–**i**) XPS spectra, (**j**) FT-IR spectra, and (**k**) UV–Vis absorbance spectra of UiO-66-NH_2_, UiO-66-NH_2_@CNT 5 wt%, and UiO-66-NH_2_@CNT 10 wt%.

Figure 5 illustrates the chemical-state evolution of UiO-66-NH_2_ upon CNT incorporation. For UiO-66-NH_2_, the XPS O 1s spectrum in Figure 5a exhibits peaks at 530.7 eV (Zr–O) and 531.8 eV (C=O), corresponding to zirconium–oxygen and carbonyl species, respectively. The C 1s spectrum in Figure 5b shows contributions from C–C/C–H (284.2 eV), C–N (285.2 eV), and C=O (288.5 eV). The N 1s spectrum in Figure 5c contains components assigned to C–N (399.1 eV) and –NH_2_ (400.1 eV), confirming the presence of amine-functionalized linkers [27]. After incorporation of CNTs (5 wt%), the N 1s spectrum (Figure 5f) shows increased N-containing contributions, including enhanced higher-binding-energy components that may be associated with protonated or interacting amine groups (–NH^3+^ or N–C/O interactions). Concurrently, an additional oxygen-related component appears in the O 1s region (Figure 5d), and the C 1s spectrum (Figure 5e) exhibits increased C–N contributions. These changes suggest interfacial electronic interactions and possible hydrogen-bond-type coupling between the amine groups of UiO-66-NH_2_ and oxygen-containing functionalities on the CNT surfaces.

At higher CNT loading (10 wt%), the MOF remains largely preserved (Figure 5g–i), while N- and O-related interfacial components become relatively more pronounced. These features are consistent with partial interfacial anchoring of UiO-66-NH_2_ particles onto CNT surfaces during in situ growth and indicate heterogeneous interfacial association rather than simple post-synthetic physical mixing. Although definitive molecular-level interactions cannot be established from the present data, such interfacial proximity may enhance contact between the photothermal and catalytic domains within the hybrid system.

As shown in Figure 5j, characteristic FTIR bands at approximately 1573 cm^−1^ (N–H bending) and 500–540 cm^−1^ (Zr–O stretching vibration of the Zr–oxo clusters) confirm preservation of the MOF [18,23,29]. The band in the 600–800 cm^−1^ region, centered near approximately 765 cm^−1^, is assigned to aromatic ring vibrations of the benzene dicarboxylate linker rather than Zr–O stretching, consistent with previous studies on UiO-66-type materials [18,23,29].

Upon CNT hybridization, a slight enhancement of the C–N stretching band near 1389 cm^−1^ is observed; although this does not constitute direct evidence of covalent bonding, it is consistent with minor interfacial perturbations induced by CNT incorporation.

Figure 5k presents the UV–Vis absorbance spectra of UiO-66-NH_2_ and the UiO-66-NH_2_@CNT composites. Compared with pristine UiO-66-NH_2_, the CNT-incorporated samples exhibit markedly enhanced light absorption across the entire visible region, together with an extended absorption tail into the near-infrared (NIR) region. This broadband enhancement becomes more pronounced with increasing CNT content, consistent with the light-harvesting contribution of CNTs within the composite framework [37,51].

UiO-66-NH_2_@CNT (5 wt%) displays a relatively stable absorption profile over the measured wavelength range, whereas the 10 wt% CNT composites show higher overall absorbance with a less uniform baseline, particularly in the NIR region. These differences are likely associated with the increased CNT content and the morphological changes observed in the composite samples. Overall, CNT incorporation enhances broadband light absorption, while excessive loading appears to modify the optical response of the hybrid system.

The interfacial relationship between UiO-66-NH_2_ and CNTs was further examined by TEM and STEM–EDS elemental mapping (Figure S4). The images show UiO-66-NH_2_ crystals in close proximity to CNT strands, indicating spatial association between the two components. Elemental mapping of C, N, O, and Zr reveals co-localization of the MOF particles and the CNT network within the analyzed region, suggesting interfacial contact rather than complete phase. Such spatial proximity may influence local thermal interactions between CNTs and UiO-66-NH_2_ under irradiation conditions, which could potentially contribute to the observed photothermal-assisted decontamination behavior. However, direct thermal-transfer pathways at the interface were not explicitly verified in the present study.

**Figure 6:**
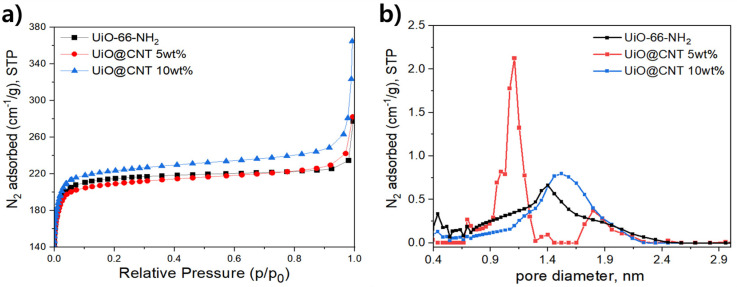
N_2_ adsorption–desorption isotherms (**a**) and pore size distributions (**b**) of UiO-66-NH_2_, UiO-66-NH_2_@CNT 5 wt%, and UiO-66-NH_2_@CNT 10 wt%.

The N_2_ adsorption–desorption isotherms and corresponding pore size distributions of UiO-66-NH_2_, UiO-66-NH_2_@CNT 5 wt%, and UiO-66-NH_2_@CNT 10 wt% are presented in Figure 6. All samples exhibit type I isotherms characteristic of predominantly microporous materials. Pristine UiO-66-NH_2_ displays a BET surface area of 859.4 m^2^ g^−1^, a total pore volume of 0.4054 cm^3^ g^−1^, and an average pore diameter of 0.94 nm, consistent with the typical microporous UiO-66 framework. Upon incorporation of 5 wt% CNTs, the BET surface area slightly decreases to 812.3 m^2^ g^−1^, while the total pore volume increases to 0.4621 cm^3^ g^−1^, which may be associated with additional interparticle voids without substantial alteration of the intrinsic framework porosity. The pore size distribution remains centered in the microporous region (~0.9 nm), supporting preservation of the intrinsic microporous structure of UiO-66-NH_2_ after CNT incorporation, along with a moderate increase in textural porosity likely originating from interfacial voids and interparticle spaces between MOF particles and CNTs.

In contrast, the UiO-66-NH_2_@CNT 10 wt% composite exhibits a higher BET surface area (892.5 m^2^ g^−1^) and total pore volume (0.5166 cm^3^ g^−1^). Because CNTs themselves do not possess intrinsic microporosity comparable to the UiO-66 framework, the increased N_2_ uptake cannot be directly interpreted as the formation of new intrinsic micropores. Additional control experiments using a physical mixture of UiO-66-NH_2_ and CNTs (10 wt%) (Figure S5) exhibited a lower BET surface area (621.9 m^2^ g^−1^), suggesting that the adsorption behavior of the composite system may not be fully explained by a simple weighted-average contribution of the individual components alone. At the same time, interparticle/textural voids are expected to mainly influence the high-relative-pressure region. Therefore, the BET increase at higher CNT loading is more conservatively interpreted as reflecting combined structural/textural effects within the composite system rather than the generation of new intrinsic micropores. Similar behavior has previously been observed in MOF/carbon composite systems [33].

In contrast, the UiO-66-NH_2_@CNT 10 wt% composite exhibits a higher BET surface area (892.5 m^2^ g^−1^) and total pore volume (0.5166 cm^3^ g^−1^). Because CNTs themselves do not possess intrinsic microporosity comparable to the UiO-66 framework, the increased N_2_ uptake is more likely associated with interfacial/textural voids and modified particle packing at higher CNT loadings rather than the formation of new intrinsic MOF micropores. Similar behavior has previously been observed in MOF/carbon composite systems [33]. However, the increased BET surface area does not necessarily indicate improved accessibility to catalytically active microporous sites. The broader pore size distribution indicates a less uniform pore environment that may result in less homogeneous mass-transport pathways despite the higher overall porosity [9]. Overall, moderate CNT loading (5 wt%) preserves the intrinsic microporous characteristics of UiO-66-NH_2_ while introducing additional textural porosity, which is consistent with the comparatively improved solvent-free decontamination performance observed for the 5 wt% composite.

**Figure 7:**
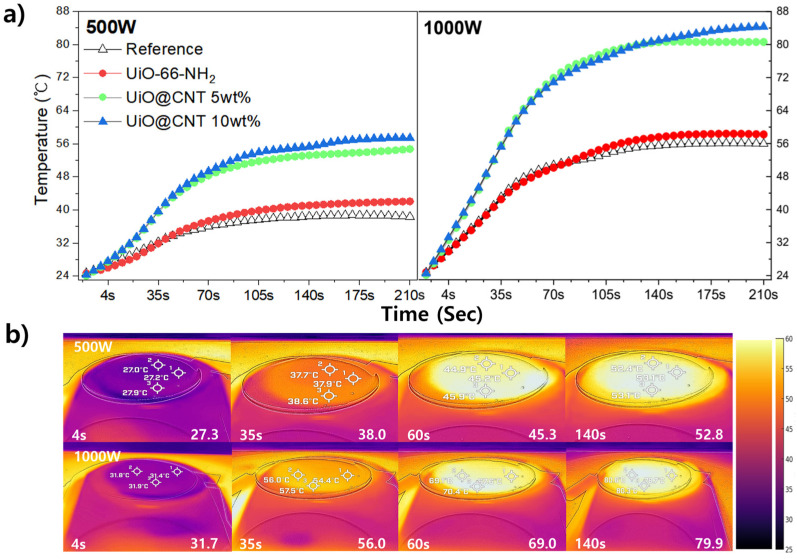
Photothermal performance of UiO-66-NH_2_ and UiO-66-NH_2_@CNT composites. (**a**) Real-time temperature profiles under 500 and 1000 W m^−2^ irradiation, showing enhanced photothermal response with CNT incorporation. (**b**) Infrared thermographic images demonstrating rapid heat generation and higher surface temperatures for the UiO-66-NH_2_@CNT samples.

As shown in the temperature profiles in Figure 7a, all samples exhibit a rapid temperature increase within the first 60 s, with the equilibrium temperature strongly dependent on CNT content and light intensity. At 500 W m^−2^, the maximum temperatures reach approximately 45 °C for UiO-66-NH_2_@CNT 5 wt% and 48 °C for UiO-66-NH_2_@CNT 10 wt%, compared with 38 °C for pristine UiO-66-NH_2_. Under stronger irradiation (1000 W m^−2^), the composite samples achieve significantly higher steady-state temperatures, approximately 73 °C for UiO-66-NH_2_@CNT 5 wt% and 80 °C for UiO-66-NH_2_@CNT 10 wt%, whereas the pristine MOF remains below 60 °C. The corresponding thermal images in Figure 7b further confirm the enhanced photothermal conversion efficiency of the CNT-containing composites. The improved light-to-heat conversion is attributed to the strong optical absorption of CNTs and the intimate interfacial contact between the CNT network and the UiO-66-NH_2_ framework, which may contribute to enhance photothermal heat generation and dissipation throughout the hybrid structure [40]. These results demonstrate that CNT incorporation enables pronounced sunlight-driven heating behavior, which is advantageous for accelerating solvent-free decontamination reactions.

## 4. Discussion

**Figure 8:**
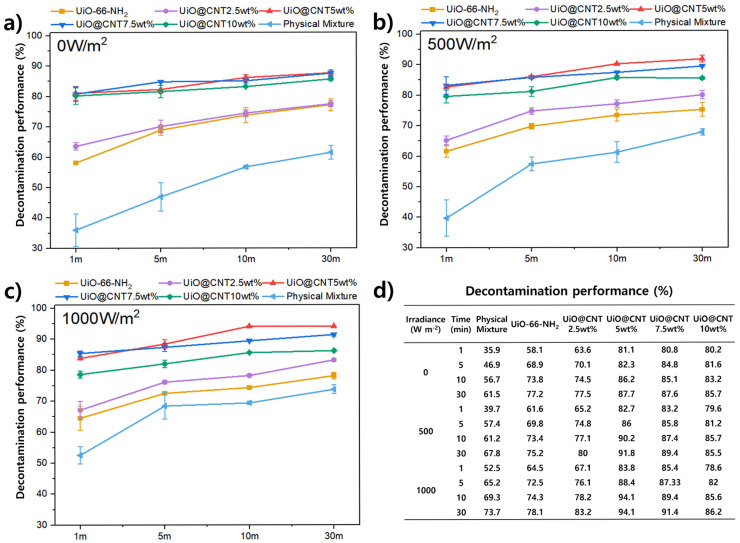
DMMP decontamination performance of UiO-66-NH_2_ and UiO-66-NH_2_@CNT composites with different CNT loadings under simulated solar irradiation: (**a**) 0 W m^−2^, (**b**) 500 W m^−2^, (**c**) 1000 W m^−2^, and (**d**) summary of DMMP removal efficiencies. The 5 wt% CNT composite exhibits the best overall performance, indicating a favorable balance between photothermal enhancement and reactive-site accessibility.

The decontamination performance of UiO-66-NH_2_ and UiO-66-NH_2_@CNT composites with varying CNT loadings (2.5, 5, 7.5, and 10 wt%) was evaluated under simulated sunlight at intensities of 0, 500, and 1000 W m^−2^. As shown in Figure 8, all samples exhibit a gradual increase in DMMP removal efficiency with reaction time, while the CNT-containing composites consistently outperform pristine UiO-66-NH_2_, indicating a positive contribution from CNT incorporation.

Under dark conditions (0 W m^−2^), UiO-66-NH_2_@CNT 5 wt% and UiO-66-NH_2_@CNT 10 wt% both achieve efficiencies above 80% within 30 min, indicating that intrinsic adsorption and catalytic activity of the UiO-66-NH_2_ framework substantially contribute to the overall DMMP removal behavior even in the absence of photothermal activation. The enhanced performance of the hybrid systems under non-irradiated conditions may additionally be associated with modified interfacial contact and accessibility of reactive sites within the composite structure.

Upon light irradiation, decontamination efficiencies further increase due to photothermal heating. At 500 W m^−2^, UiO-66-NH_2_@CNT 5 wt% reaches 91.8% efficiency after 30 min, outperforming both the lower loading (2.5 wt%, 80.0%) and higher loading samples (7.5 wt%, 89.4%; 10 wt%, 85.5%). Under stronger irradiation (1000 W m^−2^), rapid decontamination is observed, and UiO-66-NH_2_@CNT 5 wt% achieves a maximum efficiency of approximately 94% within 10–30 min, whereas further CNT addition results in slightly reduced performance.

It should also be noted that all experiments were conducted using the same total composite mass (5 mg per reaction). Accordingly, the effective UiO-66-NH_2_ contents in the UiO@CNT-5 wt% and UiO@CNT-10 wt% composites were approximately 4.75 mg and 4.50 mg, respectively. A qualitative normalization based on the nominal UiO-66-NH_2_ fraction was additionally considered under 1000 W m^−2^ irradiation conditions (Table S1), suggesting that the observed performance trends likely arise from multiple coupled factors rather than catalyst loading differences alone.

These results suggest that CNT incorporation contributes to enhanced photothermal behavior under irradiation conditions, while the overall DMMP removal performance is additionally influenced by reactive-site accessibility and interfacial/textural effects within the hybrid structure. Nevertheless, an apparent optimum CNT loading (~5 wt%) is observed under the present experimental conditions. Low CNT content (2.5 wt%) may provide limited photothermal contribution, whereas excessive CNT loading (≥7.5 wt%) may reduce reactive-site accessibility and alter the composite structure. Consequently, the 5 wt% composition provides the most balanced overall performance among the investigated systems. The comparatively lower performance of the physical mixture further suggests a possible contribution from interfacial proximity between UiO-66-NH_2_ and CNTs within the hybrid structure.

To further quantify the reaction kinetics, the decontamination behavior of the UiO-66-NH_2_@CNT (5 wt%) sample under 1000 W m^−2^ irradiation in the initial stage (1–5 min) can be reasonably described by pseudo-first-order kinetics, with an apparent rate constant of ~0.17 min^−1^. In contrast, pristine UiO-66-NH_2_ exhibits a significantly lower apparent rate, supporting a possible contribution from CNT-assisted photothermal activation during the early-stage reaction period.

To clarify the intrinsic role of CNTs, standalone CNT performance was evaluated under identical conditions (Figure S6). Pristine CNTs exhibit limited decontamination efficiency but strong photothermal heating under light irradiation, indicating that CNTs primarily function as photothermal agents rather than catalytic sites. These observations suggest that the primary catalytic contribution originates from UiO-66-NH_2_, while CNTs mainly function as photothermal components under irradiation conditions.

Despite the observed shift in the optical absorption edge upon CNT incorporation (Figure S7), the enhanced removal performance is more closely associated with photothermal heating than with electronic band structure effects. Additional dark-chamber experiments conducted at a comparable temperature (~55 °C), corresponding to the surface temperature achieved under 500 W m^−2^ irradiation, showed substantial DMMP removal efficiencies that were comparable to, although slightly lower than, those obtained under photothermal irradiation conditions (Table S2). These results suggest that thermal effects contribute significantly to the overall DMMP removal behavior, while the photothermal operating conditions may provide a modest enhancement under the present experimental conditions.

The operational stability of the UiO-66-NH_2_@CNT hybrids was evaluated through repeated decontamination cycles under solar-simulator irradiation. As shown in Figure S8, all CNT-containing composites maintain comparable decontamination efficiencies over three consecutive cycles. For example, UiO-66-NH_2_@CNT 5 wt% achieves approximately 87.0%, 88.1%, and 90.4% removal at 30 min during the first, second, and third cycles, respectively. Similar behavior is observed for the 2.5 wt% and 7.5 wt% compositions, with variations remaining within approximately ±5% across cycles. Notably, no monotonic decline in performance is detected, indicating stable catalytic functionality and photothermal responsiveness under repeated irradiation.

To assess photothermal behavior under realistic environmental conditions, outdoor temperature measurements were conducted under natural winter sunlight with an irradiance of approximately 990 W m^−2^ (Figure S9). Despite irradiance comparable to that of the solar simulator, the absolute temperature increase (~32–33 °C) is lower than that observed under laboratory irradiation (~80 °C), which can be attributed to lower ambient temperature and enhanced convective heat dissipation under open-air conditions. Importantly, the CNT-containing hybrids consistently exhibit a distinct temperature increase relative to pristine UiO-66-NH_2_ and the reference substrate, demonstrating effective photothermal conversion under natural sunlight.

Collectively, these results demonstrate that the UiO-66-NH_2_@CNT hybrids exhibit stable decontamination performance over repeated irradiation cycles and retain photothermal activity under natural sunlight, supporting their potential applicability in practical sunlight-driven systems. Although substantial DMMP removal was also observed under non-irradiated conditions, indicating contributions from intrinsic adsorption and catalytic activity of UiO-66-NH_2_, photothermal irradiation provided additional enhancement in the early-stage removal kinetics. This accelerated initial reduction in the parent simulant may be practically meaningful for rapid mitigation of highly toxic organophosphorus species.

In the FT-IR spectrum of pristine UiO-66-NH_2_ (Figure S10), characteristic bands of intact DMMP are observed at approximately 1250 cm^−1^ (P=O stretching), 1050 cm^−1^ (C–O stretching of the P–O–CH_3_ group), and 900 cm^−1^ (P–CH_3_ deformation). After decontamination, the intensities of these ester-related bands are markedly reduced, indicating loss of the intact DMMP molecular signature at the surface. Concurrent spectral modifications appear near 1000 cm^−1^ and in the low-frequency region (~710 cm^−1^), consistent with changes in the local phosphate coordination environment rather than preservation of the original ester structure; similar features have been reported in systems where ester bond cleavage produces phosphonate-like surface coordination environments [61]. Pyridine-adsorbed FT-IR analysis (Figure S11) further shows slight spectral changes near 1450 and 1600 cm^−1^ after pyridine exposure, qualitatively supporting the presence of accessible Lewis-acidic Zr sites in UiO-66-NH_2_. These observations are consistent with surface-mediated transformation of DMMP involving accessible Lewis-acidic sites rather than simple physisorption.

## 5. Conclusions

The UiO-66-NH_2_@CNT composites developed in this study demonstrate a photothermal-assisted reactive adsorption strategy for solvent-free removal of DMMP. By integrating the Lewis-acidic catalytic sites of UiO-66-NH_2_ with the efficient solar-to-heat conversion capability of CNTs, the optimized 5 wt% hybrid achieves rapid decontamination through photothermally enhanced surface reactions without the use of solvents, buffers, or external heating.

This solvent-free, powder-based approach minimizes secondary liquid waste, corrosion risks, and cross-contamination, thereby reducing hazards associated with conventional liquid-phase decontamination systems. Compared with pristine UiO-66-NH_2_, the UiO-66-NH_2_@CNT (5 wt%) composite exhibits enhanced decontamination performance under simulated sunlight while preserving the intrinsic framework structure of the MOF and maintaining a uniform photothermal response.

Overall, this work presents a practical and environmentally responsible route toward field-deployable decontamination of organophosphate simulants and provides a general design strategy for integrating photothermal and catalytic functionalities in MOF–carbon hybrid systems for sustainable hazardous chemical mitigation.

## Figures and Tables

**Table 1:** Comparison of the decontamination performance of the UiO-66-NH_2_@CNT hybrid with representative MOF-based systems for organophosphorus nerve agent simulants (DMMP and DMNP) under various conditions.

Material	Simulant	Condition	Decontamination Time/Efficiency	Form	Key Features	Ref.
UiO-66-NH_2_@CNT (5 wt%) (this work)	DMMP	Solvent-free, solar-simulated, 1000 W m^2^	≈94% in 10 min	Powder	Solvent-free, no waste, photothermal self-heating	–
Pristine UiO-66-NH_2_	DMMP/DMNP	Buffered solution	>30–60 min	Powder	Baseline hydrolysis, moisture-dependent	[35]
UiO-66/NU-1000	DMNP	Solid-phase, varying moisture	Slow (hours), improved with water	Powder	Moisture effect critical for hydrolysis	[35]
Graphene/UiO-66-NH_2_ fabric	DMNP	Photothermal (solar simulated)	Full in 20 min, reusable (>92% after 5 cycles)	Fabric	Fast photothermal response, fabric form	[59]
PDA@UiO-66-NH_2_ fabric	DMNP	NIR/solar light	Half-life ≈ 1–2 min	Fabric	Strong photothermal effect, core–shell structure	[38]
Metal oxides (e.g., MgO-based)	DMMP	Vapor-phase, high temperature	Variable, often slow	Powder	Heterogeneous, requires high temperature	[48]
Zr-MOFs (e.g., MOF-808)	Organophosphates	Buffered or humid	Fast in solution, slow in the solvent-free state	Powder	High activity in wet conditions	[20]
Various Zr-MOFs (review)	CWAs/simulants	Mostly buffered	Minutes to hours	Powder	Comprehensive, predominantly solution-based	[11]

## Data Availability

The data supporting the findings of this study are available from the corresponding author upon reasonable request. Some data are not publicly available due to ongoing research activities and potential confidentiality considerations.

## Supplementary Materials

The following supporting information can be downloaded at: https://www.mdpi.com/article/10.3390/nano16110690/s1, Figure S1: Thermogravimetric analysis (TGA) of representative DRY and humidity-conditioned (WET) UiO-66-NH_2_ samples. The humidity-conditioned sample exhibits substantially increased low-temperature mass loss below 100 °C, indicating the presence of physically adsorbed water introduced during the 75% RH conditioning process prior to the decontamination experiments; Figure S2: Energy-dispersive X-ray spectroscopy (EDS) mapping and elemental analysis of UiO-66-NH_2_, UiO-66-NH_2_@CNT 5 wt%, and UiO-66-NH_2_@CNT 10 wt%; Figure S3: Thermogravimetric analysis (TGA) curves of UiO-66-NH_2_, UiO-66-NH_2_@CNT 5 wt%, and UiO-66-NH_2_@CNT 10 wt%, measured up to 900 °C.; Figure S4: TEM and STEM–EDS elemental mapping of the UiO-66-NH_2_@CNT hybrid. (a) Bright-field TEM image showing a UiO-66-NH_2_ crystal in proximity to CNT strands. (b) STEM image of the same region. (c) Composite elemental map. (d–g) Individual elemental maps of C, N, O, and Zr, demonstrating the interfacial integration between CNTs and UiO-66-NH_2_ particles formed during in situ interfacial growth; Figure S5: N_2_ adsorption–desorption isotherms (a) and pore size distributions (b) of the physical mixture of UiO-66-NH_2_ and CNTs containing 10 wt% CNTs; Figure S6: Standalone performance of pristine CNTs under dry-state conditions. (a) DMMP decontamination efficiency of pristine CNTs under different light irradiation intensities. (b) Photothermal temperature profiles of pristine CNTs under light irradiation, showing strong heat-generation capability but limited decontamination performance; Figure S7: UV–Vis diffuse reflectance spectra and corresponding Tauc plots of pristine UiO-66-NH_2_, UiO@CNT 5 wt%, and UiO@CNT 10 wt%; Figure S8: Reusability of UiO-66-NH_2_@CNT composites (2.5, 5, and 7.5 wt%) for DMMP decontamination over three consecutive cycles under constant light irradiation (500 W m^−2^). After each cycle, the materials were dried at 60 °C before reuse. Comparable decontamination efficiencies across cycles indicate stable catalytic performance and operational robustness of the hybrid composites; Figure S9: Outdoor photothermal performance of the UiO-66-NH_2_@CNT hybrid under natural winter sunlight (ambient temperature: −1 °C; average solar intensity: 990 W m^−2^). The hybrid exhibits a measurable temperature increase compared with pristine UiO-66-NH_2_, demonstrating effective photothermal conversion under realistic environmental conditions; Figure S10: ATR-FTIR spectra of UiO-66-NH_2_ before (CTRL) and after DMMP decontamination. The pronounced attenuation of the P–CH3 (900 cm^−1^), P–O–C/C–O (1050 cm^−1^), and P=O (1250 cm^−1^) vibrational bands indicate the loss of the intact DMMP ester signature. Concurrent changes in the phosphate vibrational region suggest modifications in the local coordination environment, consistent with bond activation and transformation of the parent ester structure; Figure S11: Pyridine-adsorbed FT-IR spectra of pristine UiO-66-NH_2_ before and after pyridine exposure, showing subtle spectral changes consistent with pyridine interaction at accessible Lewis acidic sites associated with Zr-oxo nodes; Table S1: Qualitative normalization of DMMP removal efficiencies under 1000 W m^−2^ irradiation based on the nominal UiO-66-NH_2_ fraction within each UiO@CNT composite. The normalization was performed only for comparative interpretation of composite-level behavior and does not represent true site-normalized catalytic activity. (Normalized performance = Observed removal (%)/MOF fraction); Table S2: Dark-chamber decontamination test of the UiO@CNT 5 wt% composite at a temperature comparable to that achieved under 500 W m^−2^ irradiation (~55 °C).

## Abbreviations

The following abbreviations are used in this manuscript:

CWA	Chemical Warfare Agent
MOF	Metal–Organic Framework
CNT	Carbon Nanotube
DMMP	Dimethyl Methylphosphonate
DMNP	Dimethyl 4-nitrophenyl phosphate
XRD	X-ray Diffraction
SEM	Scanning Electron Microscopy
TEM	Transmission Electron Microscopy
XPS	X-ray Photoelectron Spectroscopy
FT-IR	Fourier Transform Infrared Spectroscopy
BET	Brunauer–Emmett–Teller
UV–Vis	Ultraviolet–Visible Spectroscopy
NIR	Near-Infrared
FLIR	Forward-Looking Infrared
GC-FID	Gas Chromatography–Flame Ionization Detector
PDMS	Polydimethylsiloxane
DMF	N,N-Dimethylformamide
TBP	Tributyl Phosphate
