# Supplementary material for: Photothermal-Assisted Solvent-Free Decontamination of a Nerve Agent Simulant Using UiO-66-NH2@CNT Hybrids
Source: Nanomaterials (Basel). 2026 Jun 1;16(11):690. doi: 10.3390/nano16110690 (PMC13258759; doi:10.3390/nano16110690)
Supplement: Supplementary file 1 [file nanomaterials-16-00690-s001.zip › nanomaterials-4303262-supplementary.pdf]

Photothermal-Assisted Solvent-Free Decontamination of a Nerve Agent Simulant using UiO-66-NH_2@CNT Hybrids

Supplementary Materials

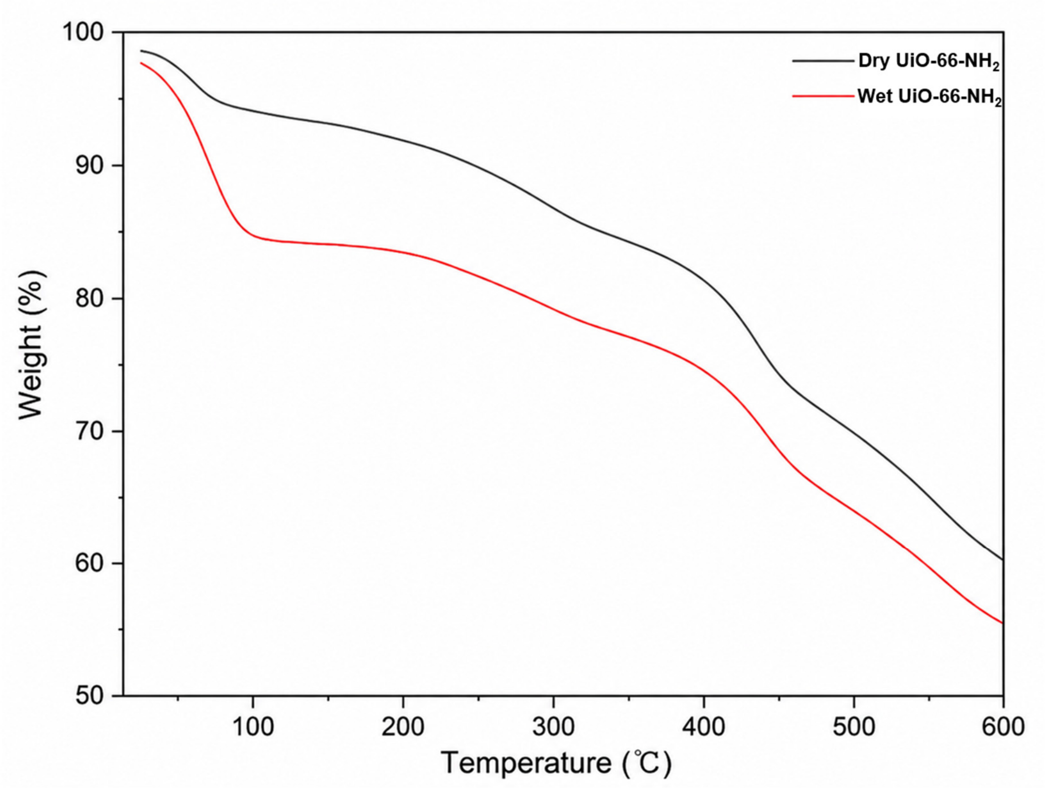

Figure S1. Thermogravimetric analysis (TGA) of representative DRY and humidity-conditioned (WET) UiO-66-NH_2 samples. The humidity-conditioned sample exhibits substantially increased low-temperature mass loss below 100 °C, indicating the presence of physically adsorbed water introduced during the 75% RH conditioning process prior to the decontamination experiments.

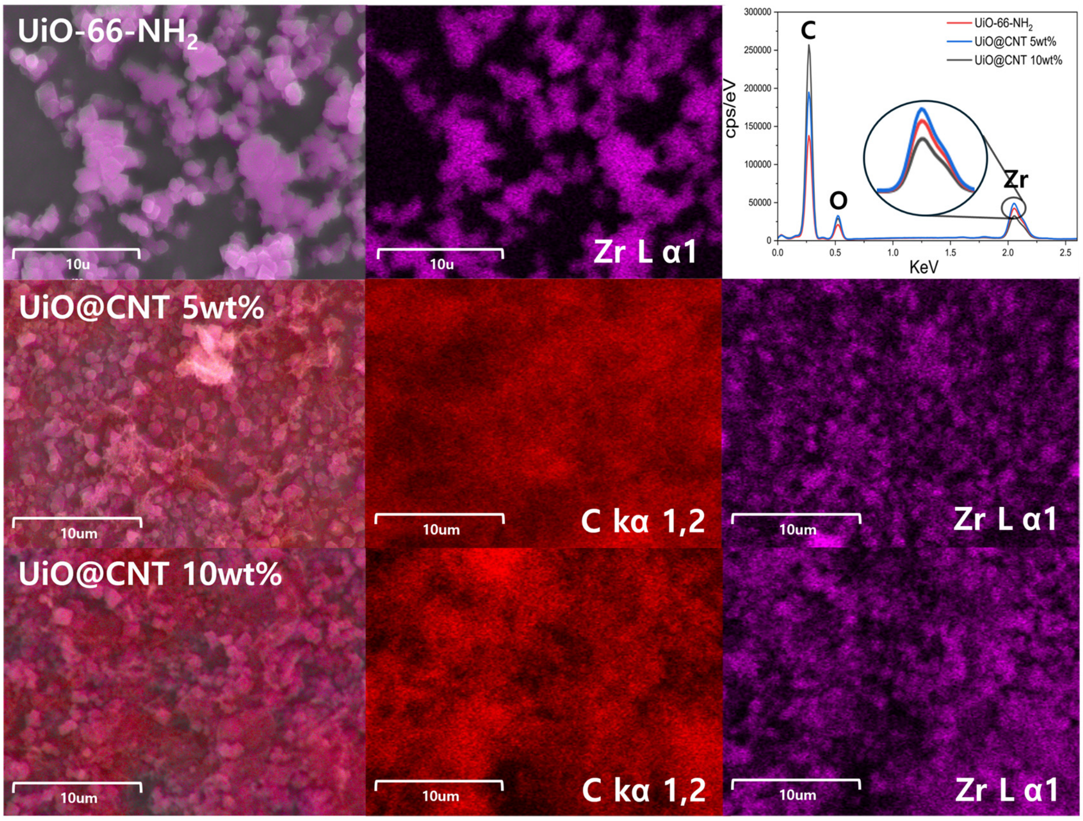

Figure S2. Energy-dispersive X-ray spectroscopy (EDS) mapping and elemental analysis of UiO-66-NH_2, UiO-66-NH_2@CNT 5 wt%, and UiO-66-NH_2@CNT 10 wt%.

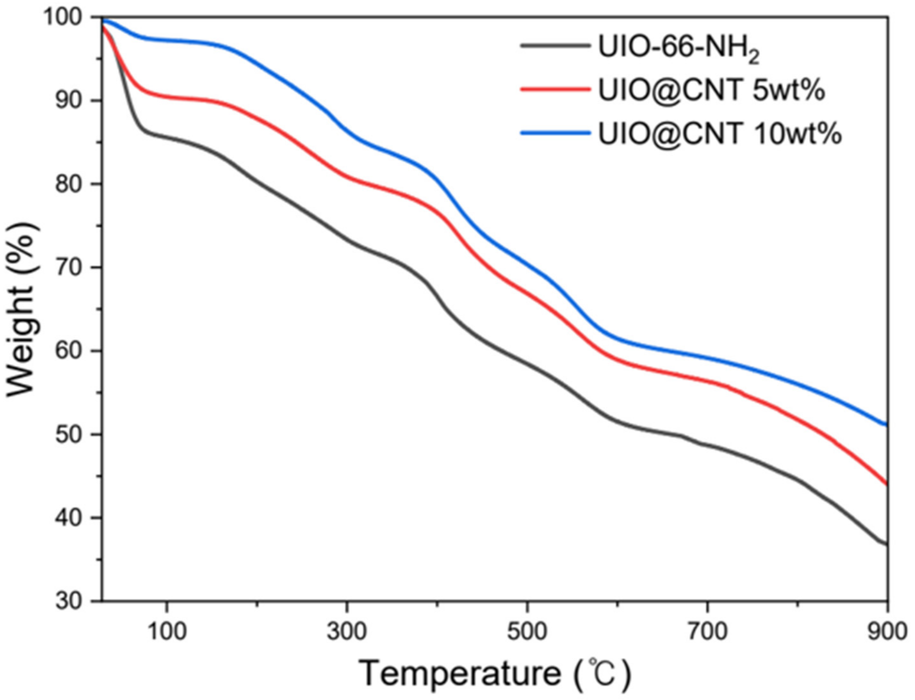

Figure S3. Thermogravimetric analysis (TGA) curves of UiO-66-NH_2, UiO-66-NH_2@CNT 5 wt%, and UiO-66-NH_2@CNT 10 wt%, measured up to 900 °C.

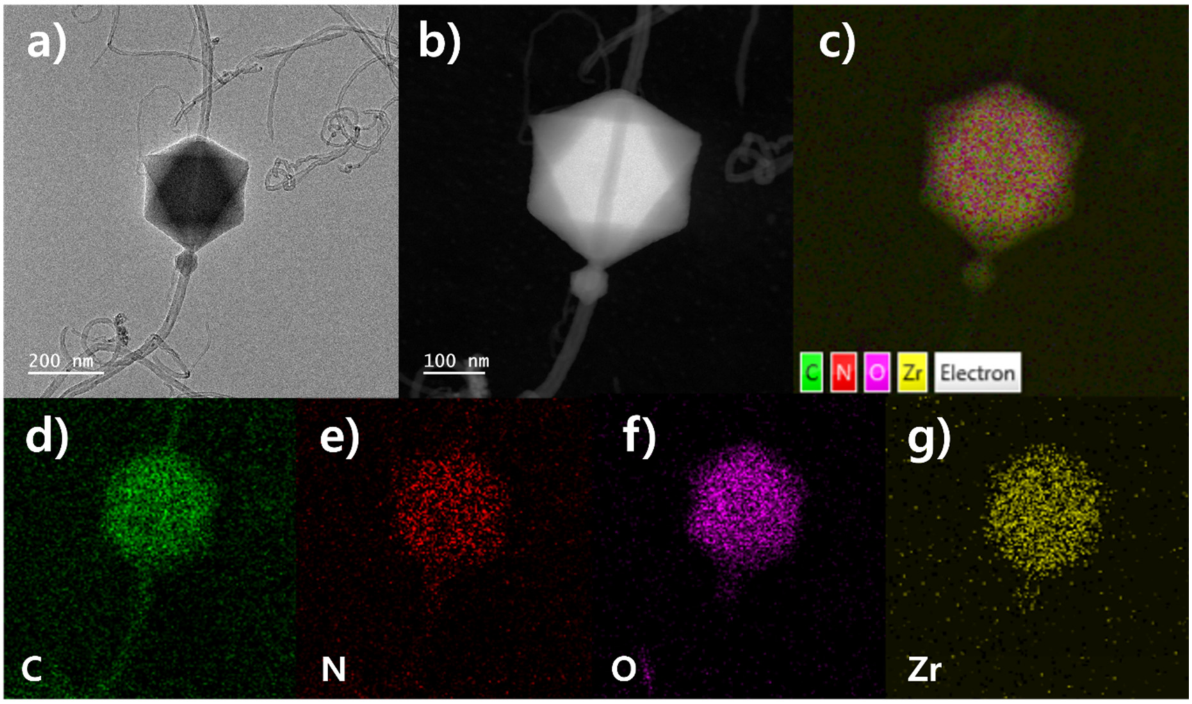

Figure S4. TEM and STEM–EDS elemental mapping of the UiO-66-NH_2@CNT hybrid. (a) Bright-field TEM image showing a UiO-66-NH_2 crystal in proximity to CNT strands. (b) STEM image of the same region. (c) Composite elemental map. (d–g) Individual elemental maps of C, N, O, and Zr, demonstrating the interfacial integration between CNTs and UiO-66-NH_2 particles formed during in situ interfacial growth.

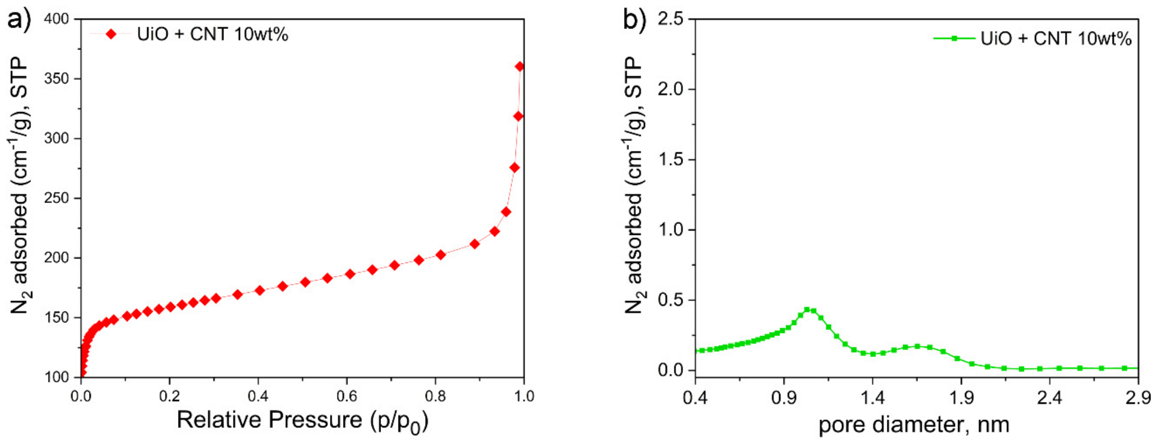

Figure S5. N_2 adsorption–desorption isotherms (a) and pore size distributions (b) of the physical mixture of UiO-66-NH_2 and CNTs containing 10 wt% CNTs.

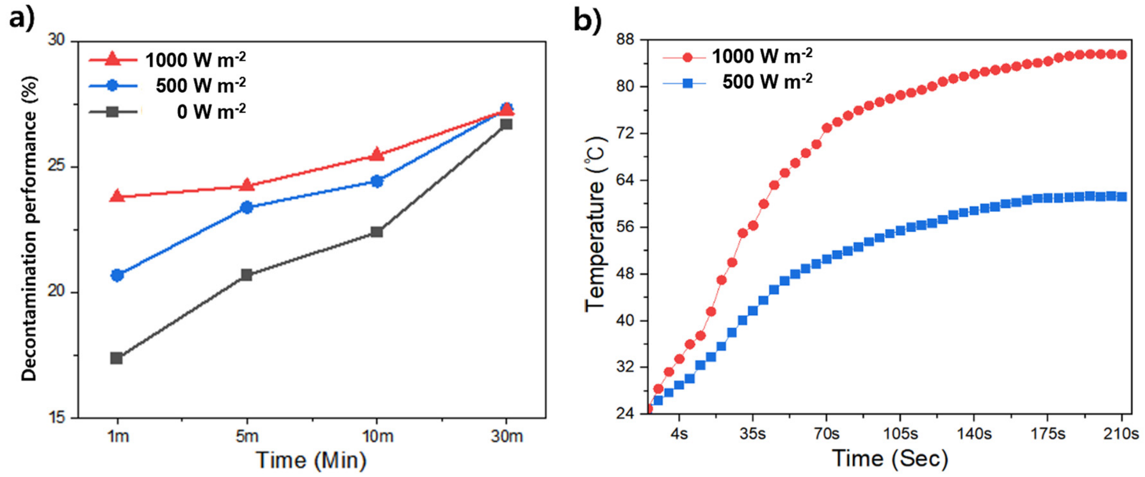

Figure S6. Standalone performance of pristine CNTs under dry-state conditions. (a) DMMP decontamination efficiency of pristine CNTs under different light irradiation intensities. (b) Photothermal temperature profiles of pristine CNTs under light irradiation, showing strong heat-generation capability but limited decontamination performance.

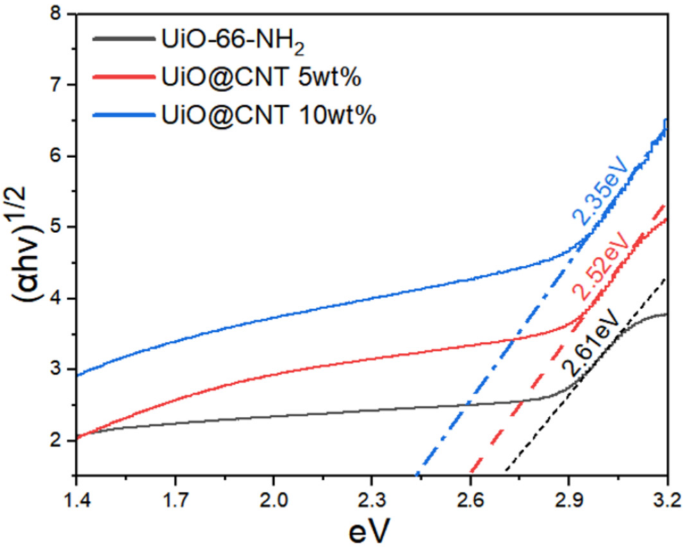

Figure S7. UV–Vis diffuse reflectance spectra and corresponding Tauc plots of pristine UiO-66-NH_2, UiO@CNT 5 wt%, and UiO@CNT 10 wt%

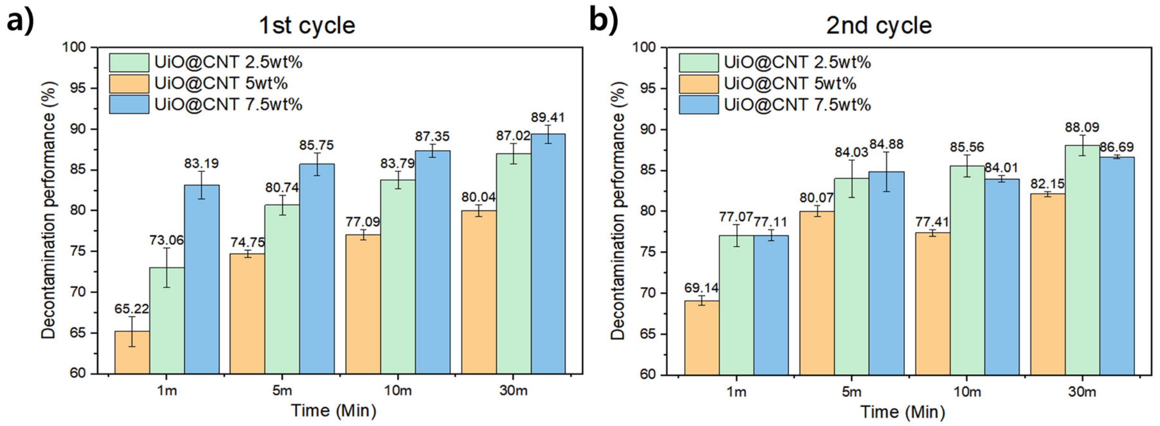

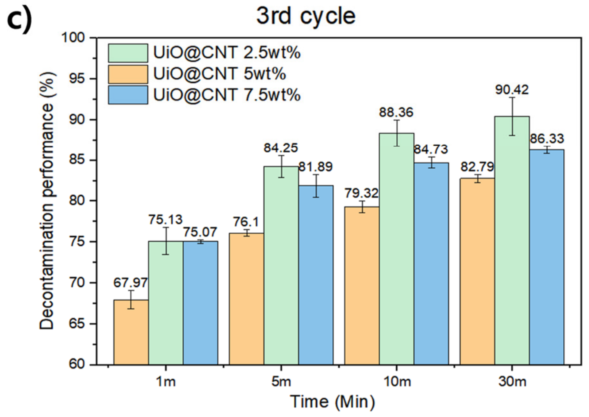

d) Purification performance (%)

Reusability	Time (min)	UiO@CNT 2.5wt%	UiO@CNT 5wt%	UiO@CNT 7.5wt%
1st	1	65.22	73.06	83.19
	5	74.75	80.74	85.75
	10	77.09	83.79	87.35
	30	80.04	87.02	89.41
2nd	1	69.14	77.07	77.11
	5	80.07	84.03	84.88
	10	77.41	85.56	84.01
	30	82.15	88.09	86.69
3rd	1	67.97	75.13	75.07
	5	76.1	84.25	81.89
	10	79.32	88.36	84.73
	30	82.79	90.42	86.33

Figure S8. Reusability of UiO-66-NH_2@CNT composites (2.5, 5, and 7.5 wt%) for DMMP decontamination over three consecutive cycles under constant light irradiation (500 W m^{-2}). After each cycle, the materials were dried at 60 °C before reuse. Comparable decontamination

efficiencies across cycles indicate stable catalytic performance and operational robustness of the hybrid composites.

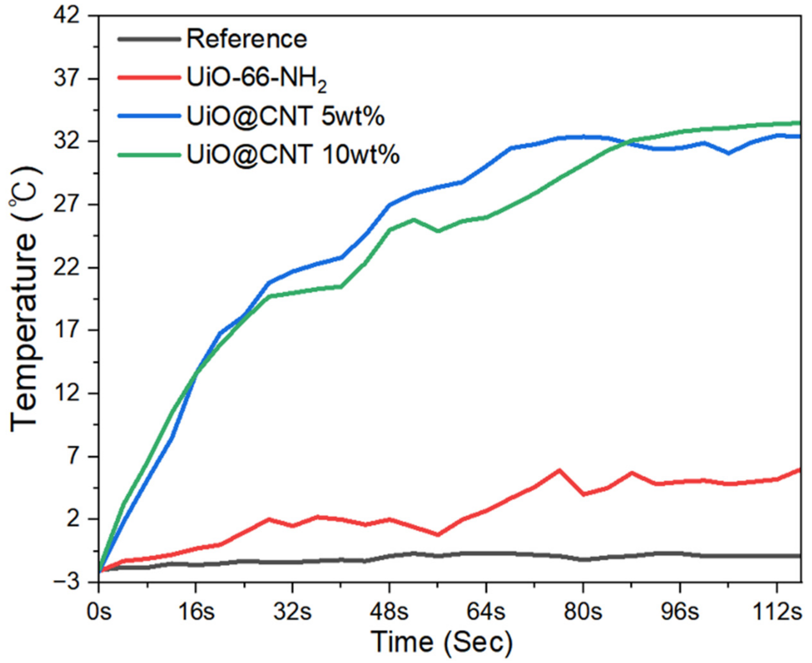

Figure S9. Outdoor photothermal performance of the UiO-66-NH_2@CNT hybrid under natural winter sunlight (ambient temperature: −1 °C; average solar intensity: 990 W m^{-2}). The hybrid exhibits a measurable temperature increase compared with pristine UiO-66-NH_2, demonstrating effective photothermal conversion under realistic environmental conditions.

.

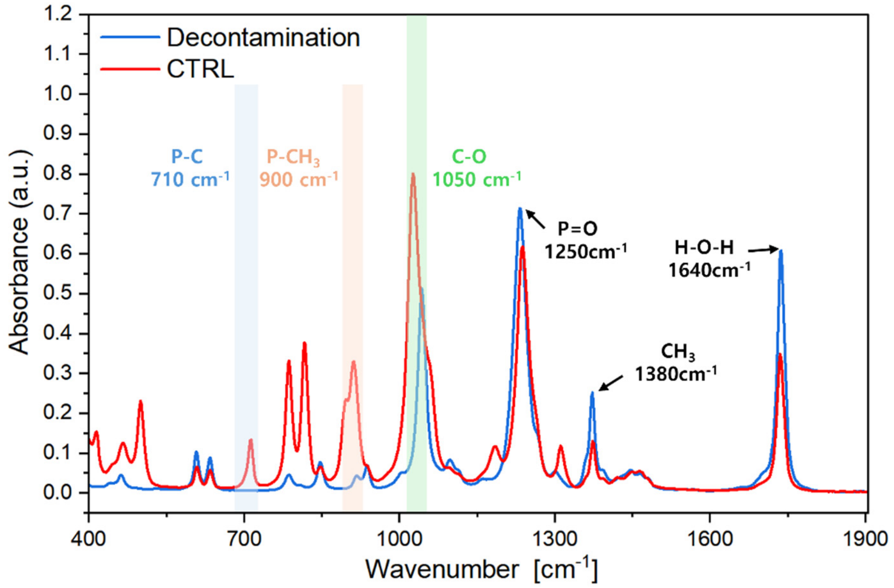

Figure S10. ATR-FTIR spectra of UiO-66-NH_2 before (CTRL) and after DMMP decontamination. The pronounced attenuation of the P–CH_3 (900 cm^{-1}), P–O–C/C–O (1050 cm^{-1}), and P=O (1250 cm^{-1}) vibrational bands indicate the loss of the intact DMMP ester signature. Concurrent changes in the phosphate vibrational region suggest modifications in the local coordination environment, consistent with bond activation and transformation of the parent ester structure.

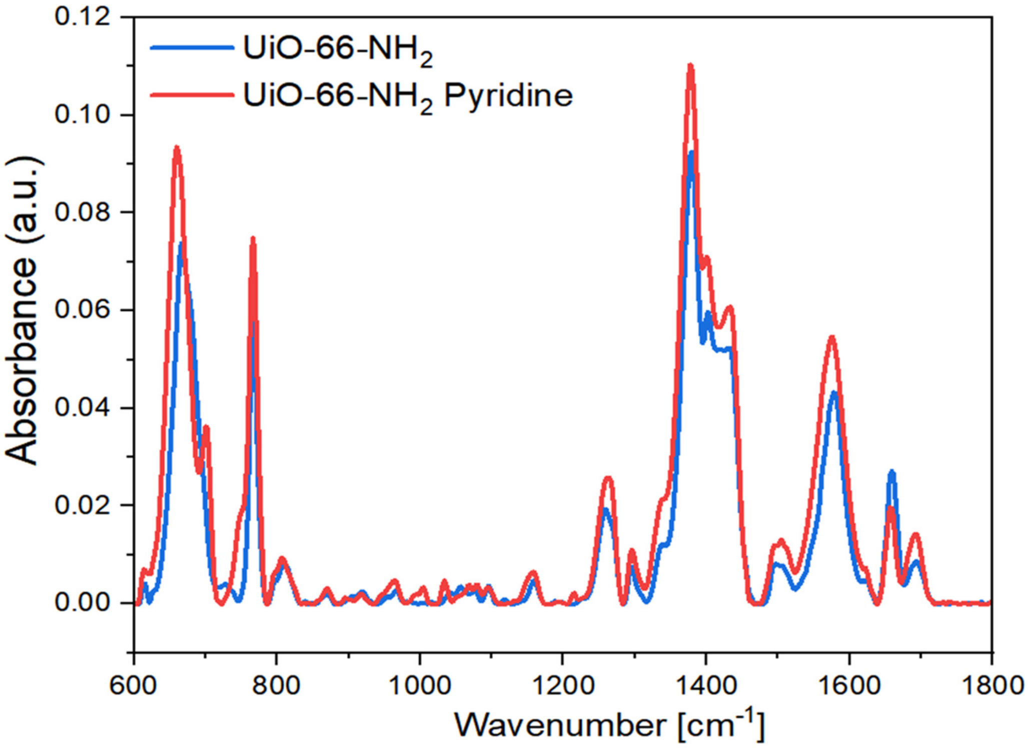

Figure S11. Pyridine-adsorbed FT-IR spectra of pristine UiO-66-NH_2 before and after pyridine exposure, showing subtle spectral changes consistent with pyridine interaction at accessible Lewis acidic sites associated with Zr-oxo nodes.

Table S1. Qualitative normalization of DMMP removal efficiencies under 1000 W m⁻² irradiation based on the nominal UiO-66-NH_2 fraction within each UiO@CNT composite. The normalization was performed only for comparative interpretation of composite-level behavior and does not represent true site-normalized catalytic activity. (Normalized performance = Observed removal (%) / MOF fraction)

Sample	Decontamination (%)	UiO-66-NH_2 fraction	Normalized performance
**UiO-66-NH_2**	74.3	1.00	74.3
**UiO@CNT 2.5 wt%**	78.2	0.975	80.2
**UiO@CNT 5 wt%**	94.1	0.950	99.1
**UiO@CNT 7.5 wt%**	89.4	0.925	96.6
**UiO@CNT 10 wt%**	85.6	0.900	95.1

Table S2. Dark-chamber decontamination test of the UiO@CNT 5 wt% composite at a temperature comparable to that achieved under 500 W m^{-2} irradiation (~55 °C).

Sample	Time(min)	Decontamination (%)
**UiO@CNT 5 wt% @ 55°C**	5	77.2
	10	81.9
	30	85.3
